# Training Specificity of Inspiratory Muscle Training Methods: A Randomized Trial

**DOI:** 10.3389/fphys.2020.576595

**Published:** 2020-12-03

**Authors:** Marine Van Hollebeke, Rik Gosselink, Daniel Langer

**Affiliations:** ^1^KU Leuven, Faculty of Movement and Rehabilitation Sciences, Department of Rehabilitation Sciences, Research Group for Rehabilitation in Internal Disorders, Leuven, Belgium

**Keywords:** training specificity, respiratory muscle training, healthy volunteers, maximal respiratory pressures, maximal inspiratory flow, lung volume specificity, pressure – flow specificity

## Abstract

**Introduction:**

Inspiratory muscle training (IMT) protocols are typically performed using pressure threshold loading with inspirations initiated from residual volume (RV). We aimed to compare effects of three different IMT protocols on maximal inspiratory pressures (PImax) and maximal inspiratory flow (V̇Imax) at three different lung volumes. We hypothesized that threshold loading performed from functional residual capacity (FRC) or tapered flow resistive loading (initiated from RV) would improve inspiratory muscle function over a larger range of lung volumes in comparison with the standard protocol.

**Methods:**

48 healthy volunteers (42% male, age: 48 ± 9 years, PImax: 110 ± 28%pred, [mean ± SD]) were randomly assigned to perform three daily IMT sessions of pressure threshold loading (either initiated from RV or from FRC) or tapered flow resistive loading (initiated from RV) for 4 weeks. Sessions consisted of 30 breaths against the highest tolerable load. Before and after the training period, PImax was measured at RV, FRC, and midway between FRC and total lung capacity (1/2 IC). V̇Imax was measured at the same lung volumes against a range of external threshold loads.

**Results:**

While PImax increased significantly at RV and at FRC in the group performing the standard training protocol (pressure threshold loading from RV), it increased significantly at all lung volumes in the two other training groups (all *p* < 0.05). No significant changes in V̇Imax were observed in the group performing the standard protocol. Increases of V̇Imax were significantly larger at all lung volumes after tapered flow resistive loading, and at higher lung volumes (i.e., FRC and 1/2 IC) after pressure threshold loading from FRC in comparison with the standard protocol (all *p* < 0.05).

**Conclusion:**

Only training with tapered flow resistive loading and pressure threshold loading from functional residual capacity resulted in consistent improvements in respiratory muscle function at higher lung volumes, whereas improvements after the standard protocol (pressure threshold loading from residual volume) were restricted to gains in PImax at lower lung volumes. Further research is warranted to investigate whether these results can be confirmed in larger samples of both healthy subjects and patients.

## Introduction

Training specificity is based on the observation that the closer the training stimulus resembles the specific characteristics of a task, the better the training outcome will be (Hawley, 2008). The principles of specificity, which are well established for locomotor muscles, have also been demonstrated for respiratory muscle training (Romer and McConnell, 2003). Similar to the force-length relationship of locomotor muscles the inspiratory pressure-volume relationship is characterized by decreasing pressure-generating capacity of the inspiratory pump with increasing lung volume (Rahn et al., 1946; Tzelepis et al., 1994b; Sieck et al., 2013). Lung volume specificity of respiratory muscle conditioning has previously been demonstrated by showing that maximal inspiratory pressures increase mostly at the specific lung volumes at which maximal efforts were performed during training (Tzelepis et al., 1994b).

Comparable to the force-velocity relationship described for locomotor muscles (Bahler et al., 1968), an inspiratory muscle pressure-flow relationship also exists which is characterized by decreases in maximal inspiratory pressures recorded at a given lung volume as inspiratory flow increase (Agostoni and Fenn, 1960). Similar to the force-velocity specificity of training for locomotor muscles (Caiozzo et al., 1981), flow specificity has also been demonstrated for inspiratory muscle training (IMT). High pressure – low flow training has been shown to mostly increase maximal inspiratory pressures, while high flow – low pressure training will mostly increase maximal inspiratory flow (V̇Imax) (Tzelepis et al., 1994a, 1999; Romer and McConnell, 2003). Most IMT protocols, both in healthy subjects and in patients are performed with pressure threshold loading (TL) with constant (isotonic) resistance throughout inspiration (Gosselink et al., 2011; Illi et al., 2012; HajGhanbari et al., 2013). Individuals are typically instructed to initiate inspirations from residual volume (RV) and to perform fast, full vital capacity inspirations (Illi et al., 2012; HajGhanbari et al., 2013) against a resistance of approximately 30-50% of maximal inspiratory pressure generating capacity (PImax) assessed at RV (McConnell and Romer, 2004; Gosselink et al., 2011; Illi et al., 2012; HajGhanbari et al., 2013). Based on the aforementioned pressure-volume relationship this constant absolute loading will gradually increase the relative load on the muscles during inspiration. Loads of 30–50% PImax, which constitute an intermediate flow and pressure stimulus when initiated at RV, will therefore gradually evolve into a high pressure/low flow stimulus at higher lung volumes. Eventually the resistance will exceed the maximal inspiratory pressure generating capacity thereby preventing further shortening and resulting in an isometric contraction. This will limit the ability to achieve full volume expansion during inspiration (Langer et al., 2015).

Initiating pressure threshold loading training from a higher lung volume, such as functional residual capacity (FRC), against a similar intermediate flow and pressure load relative to PImax assessed at FRC might be an alternative to circumvent these limitations of pressure threshold loading. Another option might be to use an alternative type of loading. In contrast to TL, tapered flow resistive loading (TFRL) does not offer a constant resistance during inspiration. After overcoming an initial threshold load at RV the external resistance will gradually reduce during inspiration. This has been shown to result in an intermediate flow and pressure load over the complete range of a full vital capacity inspiration (Langer et al., 2015).

The objective of this study was to compare lung volume specificity and pressure-flow specificity of three different IMT protocols all using intermediate flow and pressure loads. The three IMT regimens were (1) a standard protocol using TL with inspirations initiated from RV (TL-RV), (2) a protocol using TFRL with inspirations initiated from RV (TFRL-RV), and (3) a protocol using TL with inspirations initiated from FRC (TL-FRC).

Our hypotheses were that (1) TL-RV would increase PImax and V̇Imax predominantly at lower lung volumes (between RV and FRC); (2) TFRL-RV would increase both outcomes equally over the full range of vital capacity; (3) TL-FRC would increase these outcomes predominantly at higher lung volumes (between FRC and total lung capacity, TLC).

## Materials and Methods

### Subjects

Healthy volunteers were recruited via posters in the University Hospital Leuven and via public announcements on social media. Forty-eight healthy volunteers between the age of 30 and 65 years and free of cardiovascular diseases and musculoskeletal morbidity were included in the study. Spirometry was performed before the start of the intervention to ensure all volunteers had a normal pulmonary function. The local ethics committee of the University Hospitals Leuven approved this study (study number: S60754) in accordance with the ICH-GCP (international conference on harmonization guidelines on good clinical practice) principles and with the most recent version of the Helsinki Declaration. Written informed consent was obtained from all volunteers prior to participation.

### Study Design

In this parallel group study, volunteers were randomized into one of the three training groups using a computer generated randomization list (Kim and Shin, 2014). Outcome assessors were blinded to group allocation; while therapists supervising weekly training sessions and participants could not be blinded for group allocation. Sixteen volunteers performed IMT with a standard protocol using pressure threshold loading with inspirations initiated from RV (TL-RV), 16 volunteers performed IMT with a protocol using TFRL with inspirations initiated from RV (TFRL-RV), and 16 volunteers performed IMT with a protocol using pressure threshold loading with inspirations initiated from FRC (TL-FRC). Before and after the training period the maximal inspiratory pressure (PImax) and maximal inspiratory flow (V̇Imax) were measured at three different lung volumes, RV, FRC and midway between FRC and total lung capacity (1/2 IC). All volunteers trained for four consecutive weeks and performed three IMT sessions a day at home. Participants were advised to spread training sessions over the day (i.e., one in the morning, one around noon and one in the evening and were further instructed to perform their final training session on the evening before the outcome assessments. No modifications have been made to the methods after the trial commenced.

### Measurements

PImax was measured according to international guidelines (Laveneziana et al., 2019). Volunteers were seated and wore a nose clip. The PImax measurement was performed at least 3 times until the values varied less than 10% (Laveneziana et al., 2019). The maximum value was used for analysis and for the calculation of the predicted value of PImax from RV (Neder et al., 1999). Volunteers were asked to perform a maximal and forceful inspiration against a closed valve with a small air leak to prevent glottis closure during the measurement (Laveneziana et al., 2019). PImax was measured at RV, FRC and 1/2 IC. Measurements at RV were performed after a maximal expiration and at FRC at the end of a passive expiration during normal tidal breathing. For the measurements at 1/2 IC, the participants performed a maximal inspiration to TLC and were instructed to breathe out slowly and subsequently to perform a maximal inspiration when reaching the lung volume midway between 1/2 IC. Lung volumes were visualized with the flow-volume loop during the measurements. PImax was assessed and analyzed by Vmax 229 (Sensor medics, California, US), which also provided the flow-volume loops during the measurements. A spirometry was performed before and after the training period according to the international guidelines and analyzed by the Vmax 229, which also provides predicted values for the forced vital capacity and forced expiratory volume (Quanjer et al., 2012). V̇Imax was measured in a seated position and volunteers wore a nose clip. V̇Imax was obtained during single maximal inspiratory efforts performed against five different threshold loads: no load, 20, 30, 50, and 70% of subjects’ PImax measured at the corresponding lung volume. These assessments were also performed at the three different lung volumes (RV, FRC, and 1/2 IC). The measurements were performed at least 3 times until the values varied by less than 10%. The maximum value was used for analysis. V̇I was continuously recorded by a Fleisch pneumotachograph connected to a PNT digital platform (M.E.C medical electronic construction, Brussels, Belgium), sampled at 100 Hz by a data acquisition system (Micro1401-3, Cambridge Electronic Design Limited, Cambridge, United Kingdom) and then processed with a dedicated software package (Spike 2, Cambridge Electronic Design Limited, Cambridge, United Kingdom).

### Inspiratory Muscle Training Protocols

In the three inspiratory muscle training (IMT) protocols, one training session consisted of 30 breaths against the highest tolerable load. One training session a week was performed under supervision, the PImax was measured and used together with subjective effort reported by the volunteer and data on inspiratory tidal volume (only possible in the TFRL group) to progressively increase the external load weekly during the training period. The target volume response during the training was set at approximately 70% of the vital capacity (VC). The TFRL-RV group was instructed to exhale completely, until RV, through the electronic POWERbreathe KHP2 device (POWERbreathe international Ltd., Warwickshire, England) followed by a fast and deep inspiration against an external load of approximately 50% of their PImax measured at RV (Langer et al., 2015). The POWERbreathe KHP2 device recorded tidal volumes, power and work of breathing during training sessions. At the end of every training session, volunteers were asked to note down these parameters displayed by the electronic device in a diary. The TL-RV group was instructed to exhale completely until RV and the TL-FRC group was instructed to exhale passively during normal tidal breathing (until FRC). Both pressure threshold loading groups used either the POWERbreathe medic plus (load range 1–78 cmH_2_O, POWERbreathe international Ltd., Warwickshire, England) or the POWERbreathe medic (load range 10-90 cmH_2_O, POWERbreathe international Ltd., Warwickshire, England) device and performed deep inspirations against an external load of approximately 50% of their PImax measured either at RV (in the TL-RV group) or at FRC (in the TL-FRC group). Volunteers were asked to write down the external resistance that had to be overcome during IMT (in cmH_2_O) in a diary at the end of every training session they performed.

### Statistical Analysis

Data are expressed as mean ± SD unless specified otherwise. Between group differences in PImax and V̇Imax were compared with two-way repeated measures ANOVA and post hoc tests using Holm-Šídák corrections for multiple comparisons. Changes from pre-IMT to post-IMT in PImax and V̇Imax at RV, FRC and 1/2 IC within groups were also compared with two-way ANOVA and post hoc tests using Holm-Šídák corrections for multiple comparisons. Difference in tidal volume during IMT between groups were compared with unpaired *t*-tests. Statistical significance was met when *p* < 0.05. The relationships between potential confounders and primary outcomes were analyzed with Pearson correlation coefficients for continuous parameters and unpaired t-tests for dichotomous variables. These analyses were performed with GraphPad prism 8 (GraphPad Software, LCC). Between group comparisons were corrected for the impact of potential confounders by entering those as covariates in an Analysis of Covariance (ANCOVA). These analyses were performed with SPSS Statistics version 26 (IBM corporation). Sample size was calculated based on a previous study in which the increase in PImax at a high lung volume (midway between FRC and TLC) after (isometric) IMT at FRC (ΔPImax = 9%) was compared to isometric IMT performed at this lung volume (i.e., midway between FRC and TLC: ΔPImax = 13%) (Tzelepis et al., 1994b). Based on an effect size of 1.14 with a power of 80% at a significance level of 5% a sample size of 14 volunteers per training group were required to demonstrate this difference.

## Results

### Recruitment

**FIGURE 1:**
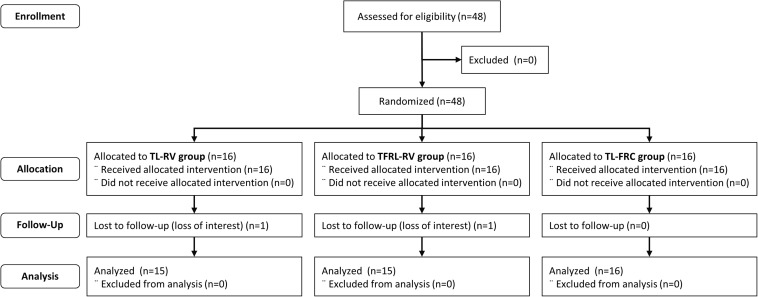
Consort diagram. TL-RV, Training protocol performed with the pressure threshold loading with inspirations initiated from residual volume; TFRL-RV, Training protocol performed with the tapered flow resistive loading with inspirations initiated from residual volume; TL-FRC, Training protocol performed with the pressure threshold loading with inspirations initiated from functional residual capacity.

A participant flow chart is provided in a diagram and depicted in Figure 1. Between 24th of January 2017 and 1st of March 2019, 48 volunteers were recruited. Trial ended when the required sample size was reached in each group. One volunteer in the TL-RV group and one volunteer in the TFRL-RV group were lost to follow-up. Therefore, data of 15 volunteers in the TL-RV group and TFRL-RV group and 16 volunteers in the TL-FRC group were analyzed (Figure 1).

### Baseline Characteristics

Baseline characteristics of the three training groups are depicted in Table 1. Baseline characteristics were similar between the training groups except for sex and PImax at baseline. In the TL-RV group, a majority of the volunteers were female, while in the TFRL-RV and the TL-FRC groups about half of the participants were female (Table 1). The mean PImax at baseline in the TFRL-RV group was slightly higher from RV and FRC as compared to the other two groups (Table 1). Lung function was considered as normal in all volunteers as both the forced vital capacity and FEV_1_/FVC ratio were above the lower limit of normal in all subjects (Quanjer et al., 2012). No adverse events were reported by the participants during the training period.

**TABLE 1:** Baseline characteristics.

	TL-RV n = 15	TFRL-RV n = 15	TL-FRC n = 16
	mean ± SD	mean ± SD	mean ± SD
Sex, n (%) Female	11 (73%)	7 (47%)	8 (50%)
Age, y	50 ± 8	47 ± 9	47 ± 9
Height, cm	171 ± 6	174 ± 9	172 ± 9
Body mass, kg	76 ± 13	72 ± 11	74 ± 12
FEV_1_, L	3.3 ± 0.8	3.8 ± 1.0	3.6 ± 0.7
% predicted	106 ± 16	115 ± 12	109 ± 13
FVC, L	4.1 ± 0.8	4.7 ± 1.3	4.5 ± 1.1
% predicted	113 ± 14	117 ± 17	114 ± 16
FEV_1_/FVC,%	79 ± 4	81 ± 5	78 ± 6
PIF, L/s	6.3 ± 2.7	5.8 ± 1.1	5.7 ± 1.4
PEF, L/s	9.2 ± 2.5	8.0 ± 1.3	8.1 ± 2.2
PImax from RV, cmH_2_O	106 ± 31	125 ± 39	100 ± 22
% predicted	114 ± 29	119 ± 31	99 ± 22
PImax from FRC, cmH_2_O	96 ± 27	105 ± 32	88 ± 21
PImax from 1/2 IC, cmH_2_O	68 ± 17	69 ± 18	66 ± 16

*Three groups performed different inspiratory muscle training protocols; TL-RV, pressure threshold loading with inspirations initiated from residual volume; TFRL-RV, Tapered flow resistive loading with inspirations initiated from residual volume; TL-FRC, Pressure threshold loading with inspirations initiated from functional residual capacity. FEV_1_, Forced expiratory volume; FVC, Forced vital capacity; FEV_1_/FVC, Tiffeneau index; PIF, Peak inspiratory flow; PEF, Peak expiratory flow; PImax, Maximal inspiratory pressure; RV, Residual volume; FRC, Functional residual capacity; 1/2 IC, midway between functional residual capacity and total lung capacity. Predicted values were derived for FEV_1_ and FVC from Quanjer et al. (2012) and for PImax from Neder et al. (1999).*

### Training Characteristics

**FIGURE 2:**
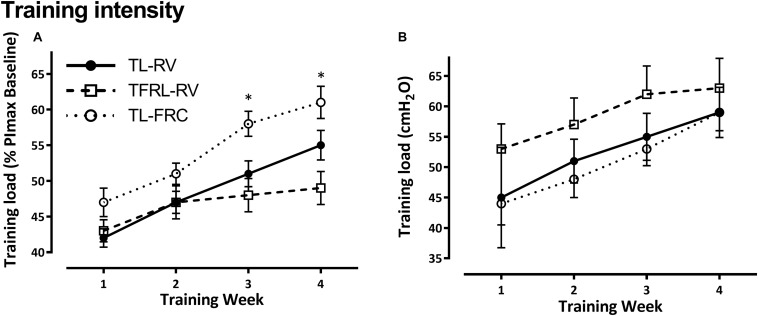
Training intensity. Evolution of training intensity throughout the 4-week inspiratory muscle training period. Over the 4 week training period, the training load was progressively increased to the highest tolerable load. In panel **(A)** the training load is expressed relative to the maximal inspiratory pressure at baseline measured at the lung volume on which the volunteers initiated the inspiration (% PImax at baseline) and in panel **(B)** the training load is depicted in absolute values in cmH_2_O. Data are expressed as mean ± SEM. PImax: maximal inspiratory pressure, TL-RV, Training protocol performed with the pressure threshold loading with inspirations initiated from residual volume; TFRL-RV, Training protocol performed with the tapered flow resistive loading with inspirations initiated from residual volume; TL-FRC, Training protocol performed with the pressure threshold loading with inspirations initiated from functional residual capacity. *p-value ≤ 0.05 indicates the significant differences in training intensities between the training groups.

**FIGURE 3:**
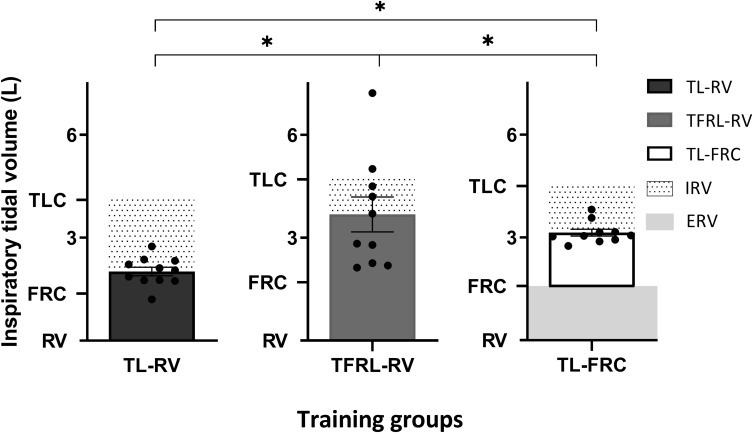
Volume response during inspiratory muscle training. In a subgroup of volunteers, the tidal volume during the three inspiratory muscle training protocols relative to the maximal reachable volume is depicted in the graph. Tidal volume in liters (mean ± SEM) is depicted per training group in individual bar charts with error bars. IRV, the mean inspiratory reserve capacity is depicted in the dotted columns and ERV, mean expiratory reserve capacity is depicted in the background shaded gray column. TL-RV (*n* = 11), Training protocol performed with the pressure threshold loading with inspirations initiated from residual volume; TFRL-RV (*n* = 10), Training protocol performed with the tapered flow resistive loading with inspirations initiated from residual volume; TL-FRC (*n* = 10), Training protocol performed with the pressure threshold loading with inspirations initiated from functional residual capacity; *p ≤ 0.05.

The patients in the TL-RV group reported on average a completion of 89 ± 3% of planned training sessions and the TL-FRC group reported a completion of 86 ± 1%. The adherence in these two groups was significantly higher than the adherence of 79 ± 4%, objectively recorded by the training device, in the TFRL-RV group (TFRL-RV vs TL-RV; *P* = 0.002, TFRL-RV vs TL-FRC; *P* = 0.01). Training intensity progressively increased over the 4-week training period in the three training groups (Figure 2). The training intensity expressed as percentage of the PImax assessed from the lung volume on which IMT was initiated, was significantly higher in the TL-FRC group in the last two weeks of the training period compared to the TL-RV group (week 3: *P* < 0.001 and week 4: *P* = 0.02) and the TFRL-RV group (week 3: *P* < 0.001 and week 4: *P* = 0.06). However, when training intensity is expressed in absolute values (cmH_2_O) the intensity was higher, although not significantly, in the TFRL-RV group in comparison to the other two groups. The volume responses during the training were significantly different between the three groups (Figure 3). The TL-RV group performed the IMT with a significant lower mean tidal volume compared to the TFRL-RV group (2.0 ± 0.4L vs 3.2 ± 1.6L; *P* = 0.03) and covered 49% of the VC in the TL-RV group and 79% of the VC in the TFRL-RV group (Figure 3). The volume response in the TL-FRC was the lowest of the three training groups with on average 1.6 ± 0.32L (TL-RV vs TL-FRC; *P* = 0.01 and TFRL-RV vs TL-FRC; *P* = 0.01) and covered approximately 54% of the inspiratory capacity (Figure 3). The remaining inspiratory reserve volume (IRV) at end inspiration during IMT-RV was the highest with 2.1L on average. TL-FRC resulted in a lower IRV of 1.4L and the TFRL-RV resulted in the lowest IRV of 1.0L (Figure 3).

#### Inspiratory Muscle Strength

**FIGURE 4:**
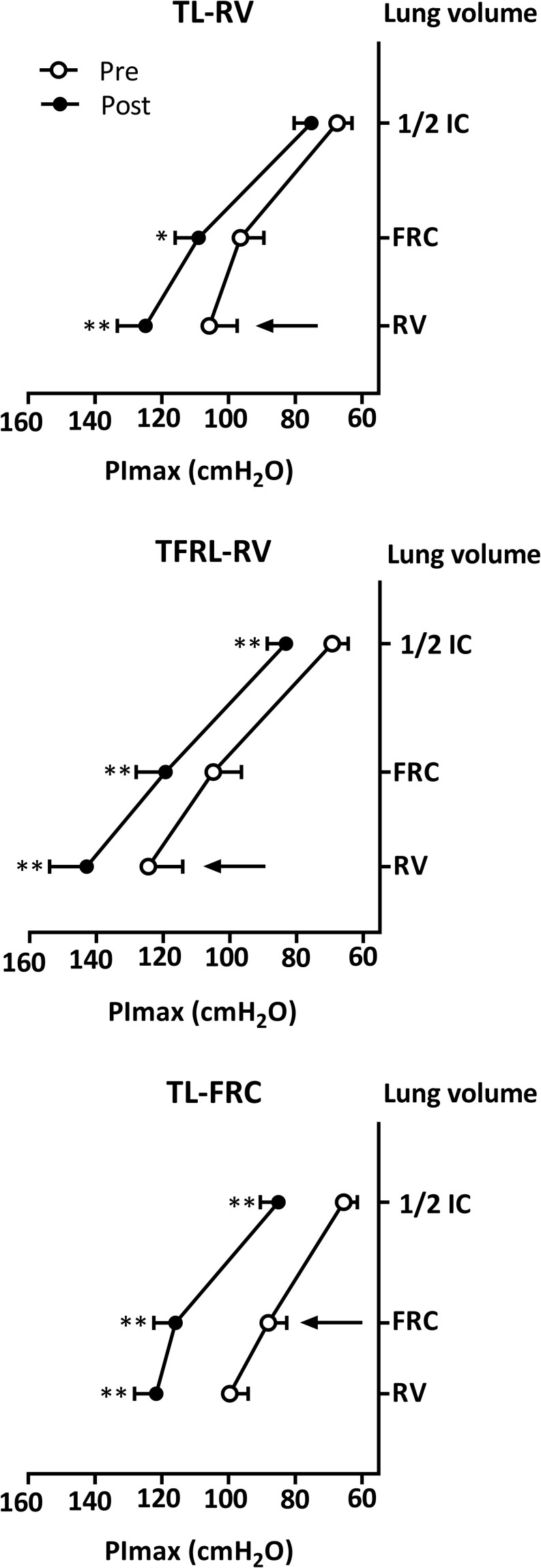
Increase in inspiratory muscle strength. Data on inspiratory muscle strength before (open circles) and after (closed circles) the 4-week training period on 3 different lung volumes are depicted in the figure, expressed as mean ± SEM. The arrow indicates the lung volume on which the inspiratory muscle training was initiated. PImax, inspiratory muscle strength; RV, Residual volume; FRC, Functional residual capacity; 1/2 IC, lung volume midway between FRC and TLC. TL-RV (*n* = 15), Training protocol performed with the pressure threshold loading with inspirations initiated from residual volume; TFRL-RV (*n* = 15), Training protocol performed with the tapered flow resistive loading with inspirations initiated from residual volume; TL-FRC (*n* = 16), Training protocol performed with the pressure threshold loading with inspirations initiated from functional residual capacity. *p-value ≤ 0.05, **p-value ≤ 0.001.

The PImax in the TL-RV increased mostly at lower lung volumes with significant increases from RV (*P* < 0.001) and from FRC (*P* = 0.01), but not from 1/2 IC (*P* = 0.09, Figure 4). Volunteers in the TFRL-RV group and TL-FRC group increased their PImax significantly from all lung volumes (Figure 4). Between group comparisons revealed that the increase of PImax from FRC in the TL-FRC group was significantly higher compared to the increase from FRC in the TL-RV group (*P* = 0.02) (see Figure 4 and Table 2). The apparent between group baseline differences in sex distribution, as well as PImax (both in cmH_2_O and in% of predicted normal value) and their role as potential confounders of the treatment effect were further investigated (see Table 3). We observed weak and non-significant correlations between these continuous variables and increases in PImax. The same was true for the adherence to the training and differences in outcomes between males and females.

**TABLE 2:** Increase in maximal inspiratory mouth pressures in cmH_2_O.

Mean ± SD	Pre	Post	*P*-value
**TL-RV**			
RV	106 ± 32	125 ± 33	<0.001
FRC	96 ± 27	109 ± 27	0.01
1/2 IC	68 ± 17	75 ± 20	0.09
**TFRL-RV**			
RV	125 ± 40	143 ± 43	0.001
FRC	105 ± 33	119 ± 34	<0.001
1/2 IC	69 ± 19	83 ± 22	<0.001
**TL-FRC**			
RV	100 ± 22	122 ± 26	<0.001
FRC	88 ± 22	116 ± 26	<0.001
1/2 IC	66 ± 16	85 ± 22	<0.001

*Data on inspiratory muscle strength before (pre) and after (post) the 4-week training period on 3 different lung volumes RV, Residual volume; FRC, Functional residual capacity; 1/2 IC, lung volume midway between FRC and TLC. TL-RV (n = 15), Training protocol performed with the pressure threshold loading with inspirations initiated from residual volume; TFRL-RV (n = 15), Training protocol performed with the tapered flow resistive loading with inspirations initiated from residual volume; TL-FRC (n = 15), Training protocol performed with the pressure threshold loading with inspirations initiated from functional residual capacity.*

**TABLE 3:** Covariate analyses.

		Increase in PImax (Δ cmH_2_O)			Increase in V̇Imax (Δ L/s)		
Descriptive analysis		Mean	95% confidence interval		Mean	95% confidence interval	
	TL-RV	13.1	8; 18.3		0.22	−0.01: 0.46	
	TFRL-RV	15.6	10.1; 21		1.07	0.79: 1.35	
	TL-FRC	23.1	17.5; 28.7		0.70	0.53: 0.8	

**Independent covariate analyses**							

**Sex**		**Increase in PImax (**Δ** cmH_2_O)**			**Increase in V̇Imax (**Δ** L/s)**		
**Unpaired t-test**		**(mean ± SD)**			**(mean ± SD)**		

	Female (*n* = 26)	16.9 ± 18.5			0.59 ± 0.62		
	Male (*n* = 20)	18 ±18.9			0.77 ±1.0		
	*p*-value	0.74			0.20		

**ANCOVA analysis**		**Mean square**	**F**	***p*-value**	**Mean Square**	**F**	***p*-value**

Tests of between subjects Effects, Sources	Group	845.83	2.54	0.08	1.03	1.93	0.15
	Sex	14.25	0.04	0.84	0.01	0.02	0.88

**Estimates**		**Mean**	**95% confidence interval**		**Mean**	**95% confidence interval**	

Covariates appearing in the model are evaluated at the following values: sex = 0.43.	TL-RV	11.9	6.1; 17.6		0.11	−0.12; 0.34	
	TFRL-RV	14.9	9.4; 20.4		1.01	0.79; 1.23	
	TL-FRC	23.2	17.9; 28.4		0.7	0.49; 0.91	

**PImax at baseline in absolute values (cmH_2_O)**		**Increase in PImax (**Δ** cmH_2_O)**			**Increase in V̇imax (**Δ** L/s)**		
**Pearson correlation**		***r***	***r*^2^**	***p*-value**	***r***	***r*^2^**	***p*-value**

		−0.09	0.01	0.29	0.14	0.02	0.11

**ANCOVA analysis**		**Mean Square**	**F**	***p*-value**	**Mean Square**	***F***	***p*-value**

Tests of between subjects Effects, Source	Group	204.0	0.61	0.55	1.30	2.50	0.09
	PImax at baseline in cmH_2_O	241.1	0.72	0.4	0.01	0.02	0.88

**Estimates**		**Mean**	**95% confidence interval**		**Mean**	**95% confidence interval**	
Covariates appearing in the model are evaluated at the following values: PImax RV Pre = 109.8.cmH_2_O	TL-RV	12.8	7.3; 18.2		0.19	−0.03; 0.41	
	TFRL-RV	15.3	9.5; 21.1		0.94	0.71; 1.17	
	TL-FRC	22.4	16.6; 28.2		0.7	0.46; 0.92	

**PImax at baseline in relative values (%pred)**		**Increase in PImax (**Δ** cmH_2_O)**			**Increase in V̇imax (**Δ** L/s)**		
**Pearson correlation**		***r***	***r*^2^**	***p*-value**	***r***	***r*^2^**	***p*-value**

		−0.14	0.02	0.11	−0.02	0.06	0.81

		**Increase in PImax (**Δ** cmH_2_O)**			**Increase in V̇Imax (**Δ** L/s)**		
**ANCOVA analysis**		**Mean Square**	***F***	***p*-value**	**Mean Square**	***F***	***p*-value**

Tests of between subjects Effects, Source	Group	41.5	0.12	0.88	0.08	0.14	0.87
	PImax at baseline in cmH_2_O	193.8	0.58	0.45	0.34	0.58	0.45

**Estimates**		**Mean**	**95% confidence interval**		**Mean**	**95% confidence interval**	

Covariates appearing in the model are evaluated at the following values: PImax RV%pred = 110.2%	TL-RV	13.3	7.8; 18.7		0.24	0.01; 0.46	
	TFRL-RV	16.3	10.7; 22		1.05	0.81; 1.28	
	TL-FRC	23	17; 29		0.66	0.41; 0.91	

**Training adherence (%completed training sessions of planned sessions)**		**Increase in PImax (**Δ** cmH_2_O)**			**Increase in V̇imax (**Δ** L/s)**		
**Correlation**		***r***	***r*^2^**	***p*-value**	***r***	***r*^2^**	***p*-value**

		−0.05	0.002	0.61	0.04	0.001	0.64

**ANCOVA analysis**		**Mean Square**	***F***	***p*-value**	**Mean Square**	***F***	***p*-value**

Tests of between subjects Effects, Source	Group	325.9	1.1	0.34	0.02	0.05	0.95
	Adherence (%completed)	308.3	1.0	0.31	0.52	0.86	0.36

**Estimates**		**Mean**	**95% confidence interval**		**Mean**	**95% confidence interval**	

Covariates appearing in the model are evaluated at the following values: Intensity = 49.6%.	TL-RV	13.5	8.3; 18.7		0.23	−0.01; 0.46	
	TFRL-RV	15.8	10.5; 21.0		1.12	0.89; 1.36	
	TL-FRC	23.5	18.0; 29.0		0.75	0.50; 0.99	

*The relationships between potential confounders and primary outcomes were analyzed with Pearson correlation coefficients for continuous parameters and unpaired t-tests for dichotomous variables. Between group comparisons were corrected for the impact of potential confounders by entering those as covariates in an Analysis of Covariance (ANCOVA). Estimated means corrected for the potential confounder and original descriptive analyses are provided in the table. PImax, maximal inspiratory pressure; V̇Imax, maximal inspiratory flow; RV, residual volume; TL-RV (n = 15), Training protocol performed with the pressure threshold loading with inspirations initiated from residual volume; TFRL-RV (n = 15), Training protocol performed with the tapered flow resistive loading with inspirations initiated from residual volume; TL-FRC (n = 16), Training protocol performed with the pressure threshold loading with inspirations initiated from functional residual capacity.*

### Maximal Inspiratory Flow

**FIGURE 5:**
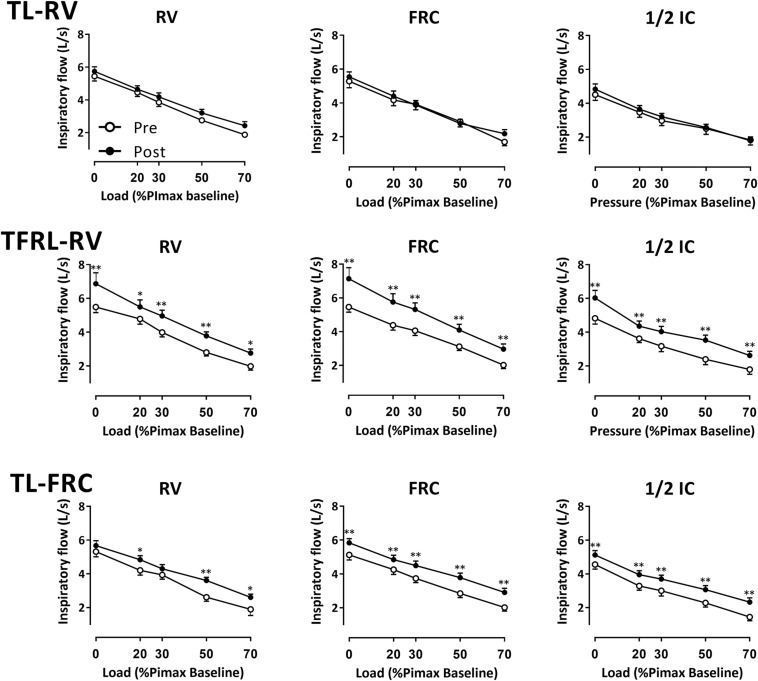
Within group comparison of increase in maximal inspiratory flow. Data on maximal inspiratory flow before (open dots) and after (closed dots) the 4-week training period on 3 different lung volumes measured against external loads performed with a pressure threshold loading: no external load, 20, 30, 50, and 70% of the PImax baseline, maximal inspiratory strength at baseline are depicted in the figure. The three panels above represent the TL-RV group (*n* = 15) in the middle the TFRL-RV group (*n* = 15) and below the TL-FRC group (*n* = 16). All measurements were performed on three lung volumes, RV, Residual volume; FRC, functional residual capacity and 1/2 IC, midway between FRC and total lung capacity. Data are presented as mean ± SEM. TL-RV, Training protocol performed with the pressure threshold loading with inspirations initiated from residual volume; TFRL-RV, Training protocol performed with the tapered flow resistive loading with inspirations initiated from residual volume; TL-FRC, Training protocol performed with the pressure threshold loading with inspirations initiated from functional residual capacity.

**FIGURE 6:**
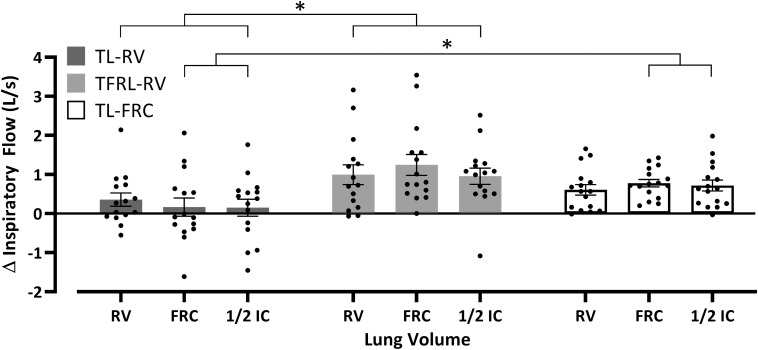
Between group comparison of the increase in maximal inspiratory flow. The average increase in maximal inspiratory flow per lung volume independently of the external loading during the inspiration. Data are depicted as mean ± SEM and significant differences between the training groups are marked with the horizontal brackets and *p ≤ 0.05. Δ Inspiratory flow, difference between post-training maximal inspiratory flow and pre-training maximal inspiratory flow; RV, residual volume; FRC, functional residual capacity; 1/2 IC, midway between FRC and total lung capacity; TL-RV (*n* = 15), Training protocol performed with the pressure threshold loading with inspirations initiated from residual volume; TFRL-RV (*n* = 15), Training protocol performed with the tapered flow resistive loading with inspirations initiated from residual volume; TL-FRC (*n* = 16), Training protocol performed with the pressure threshold loading with inspirations initiated from functional residual capacity.

V̇Imax in the TL-RV group did not increase significantly at any of the lung volumes at which the maximal inspiratory maneuvers were performed against the different external loads (Figure 5 and see Supplementary Table 1). The TFRL-RV group significantly increased V̇Imax at all the lung volumes and at all different external loadings. The TL-FRC group significantly increased V̇I at all external loadings at FRC and at 1/2 IC and against some external loadings at RV (Figure 5 and see Supplementary Table 1). No significant changes in inspiratory volume against any of the external loads at any of the lung volumes were observed in the TL-RV group and TFRL-RV group (see Supplementary Table 2). The TL-FRC group increased the inspiratory volume against 70% of the PImax at baseline at RV and at FRC and against 50% of the PImax at baseline at FRC (see Supplementary Table 2). The mean increases of V̇Imax in the TFRL-RV group ranged from 0.96L/s at both RV and 1/2 IC to 1.25L/s at FRC. Increases in V̇Imax at FRC were significantly higher in comparison to the increases at RV and 1/2 IC (both *P* = 0.01, Figure 6 and see Supplementary Table 3). The TL-FRC training group had comparable increases of V̇Imax at all lung volumes ranging from 0.61 L/s at RV to 0.78 L/s at FRC (Figure 6 and see Supplementary Table 3). The increases of V̇Imax in the TL-RV group ranged from 0.15 L/s at 1/2 IC to 0.36 L/s at RV. These changes did not reach statistical significance. The average increase of V̇Imax at every lung volume, independently from the intensity of the external load is depicted in Figure 6. The increases of V̇Imax in the TFRL-RV group were significantly higher in comparison to the TL-RV group at all lung volumes. The increases in V̇Imax in the TL-FRC group were significantly higher than in the TL-RV group at FRC and 1/2 IC, but not at RV (Figure 6 and see Supplementary Table 4). The apparent between group baseline differences in sex distribution, as well as PImax (both in cmH_2_O and in% of predicted normal value) and their role as potential confounders of the treatment effect were further investigated (see Table 3). We observed weak and non-significant correlations between these continuous variables and increases in V̇Imax during the training period. The same was true for the adherence to the training and differences in outcomes between males and females.

## Discussion

### Main Findings

In agreement with our initial hypothesis the standard protocol (TL-RV) did increase PImax at lower lung volumes (RV and FRC), but not from 1/2 IC. In contrast to our initial hypotheses, no changes in V̇Imax were observed after TL-RV, not even at lower lung volumes. As expected TFRL-RV consistently increased PImax and V̇Imax at all lung volumes. In agreement with our initial hypotheses, the alternative TL training protocol (TL-FRC) increased both PImax and V̇Imax at higher lung volumes (i.e., between FRC and TLC) but did also result in increases in these outcomes (albeit less pronounced) from RV.

### Inspiratory Muscle Strength

Our findings confirm results on volume specificity of IMT from a previous study, in which isometric training resulted in an increase of PImax predominantly at the specific lung volumes at which training was performed (Tzelepis et al., 1994b). In this study, IMT was performed 5 times a week for 6 weeks with 30 repeated maximal isometric contractions (Tzelepis et al., 1994b). When performed from residual volume this increased PImax predominantly at lower lung volumes (i.e., between RV and FRC, RV: + 43 cmH_2_O, FRC: + 21 cmH_2_O and no significant effect from 1/2 IC: + 5 cmH_2_O) (Tzelepis et al., 1994b). The TL-RV group in our study followed a similar pattern (RV: + 19 cmH_2_O, FRC: + 13 cmH_2_O and no significant effect from 1/2 IC: + 7 cmH_2_O, Figure 4 and Table 2). When IMT was performed from FRC changes in a previous study were most pronounced at this volume with significant increases in PImax also observed on lower and higher volumes (RV: + 23cmH_2_O, FRC: + 29 cmH_2_O, 1/2 IC: + 13 cmH_2_O) (Rahn et al., 1946; Tzelepis et al., 1994b). These improvements are comparable to the increases in PImax observed in our group that performed TL-FRC IMT (RV: + 22 cmH_2_O, FRC: + 28 cmH_2_O, 1/2 IC: + 19 cmH_2_O, Figure 4 and Table 2). The increases in PImax in the TFRL-RV group were equally high at all lung volumes (RV: + 18 cmH_2_O, FRC: + 14 cmH_2_O, 1/2 IC: + 14 cmH_2_O, Figure 4 and Table 2). Both TL-FRC and TFRL-RV resulted in increased PImax at higher lung volumes (between FRC and TLC, Figure 4) to a similar degree as a previous study in which isometric strength training was performed on this lung volume (1/2 IC: + 16 cmH_2_O) (Tzelepis et al., 1994b). These findings confirm that increases in PImax are volume specific and that for improvements at higher lung volumes to occur the training stimulus needs to be offered at, or in close proximity to these volumes. These observations are also in line with findings from previous studies on resistance training of locomotor muscles. Increases in strength in these studies were also highly specific to the muscle lengths at which (isometric) training was performed (Morrissey et al., 1995; Folland et al., 2005).

### Maximal Inspiratory Flow

Training in the TFRL-RV group, increased V̇Imax at all lung volumes and against all different external loads as was initially hypothesized (Figure 5 and see Supplementary Table 1). The magnitudes of increases in V̇Imax in the TFRL-RV group at RV, measured without external loading (+ 25% V̇Imax at baseline), are comparable to findings from a previous study in which TL-RV was offered at 50% PImax (+ 25% V̇Imax at baseline) (Romer and McConnell, 2003). Similar magnitudes of increases of V̇Imax after TFRL-RV IMT were observed at FRC (+ 29% V̇Imax at baseline) and at 1/2 IC (+ 25% V̇Imax at baseline, Figure 5 and see Supplementary Table 1). These findings indicate that TFRL-RV provides an intermediate pressure-flow stimulus over the full volume range. In contrast to our initial hypothesis, TL-RV did not increase maximal inspiratory flow at RV (Figure 5 and see Supplementary Table 1). With TL-RV, the increase in V̇Imax at RV was considerably smaller (no significant effect: + 6% V̇Imax at baseline) than in previous studies (Tzelepis et al., 1994a; Romer and McConnell, 2003). Smaller effects at FRC (+ 4% V̇Imax at baseline) and at 1/2 IC (+ 7% V̇Imax at baseline) were expected due to the nature of the constant threshold load which was expected to result in a high pressure – low flow stimulus at these higher volumes. These findings indicate that even at RV the TL-RV loading rather constituted a high pressure- low flow stimulus in our subjects. TL-FRC on the contrary resulted in an expected increase in V̇Imax at higher lung volumes (FRC and 1/2 IC) albeit to a lesser extent than after TFRL training. Changes in V̇Imax were significant both at FRC (+ 14% V̇Imax at baseline) and at 1/2 IC (+ 11% V̇Imax at baseline). This indicates that TL-FRC constituted an intermediate pressure-flow stimulus at higher lung volumes. As expected the increase of V̇Imax in the TL-FRC group was limited at RV (no significant effect: + 8% V̇Imax at baseline, Figure 5 and see Supplementary Table 1) since training was not performed at this volume. These observations again confirm the concept of volume specificity of training effects. Smaller effects on V̇Imax in TL-RV group are somewhat unexpected given the fact that on a weekly basis during supervised sessions participants received the same instructions on how to perform the training at home as the TL-FRC group and that training intensity (relative to PImax) was comparable between the two groups. A possible explanation could be related to the fact that during TL end expiratory lung volumes during unsupervised sessions cannot be completely controlled. This is in contrast to the TFRL training during which auditory stimuli and recorded volume responses after the training can be used to control breathing pattern during unsupervised training sessions. As a result, we hypothesize that participants in the TL-RV group might not have always initiated inspirations during unsupervised sessions after full expirations but from a somewhat higher lung volume instead. This might have resulted in a stimulus leaning more toward a high-pressure-low flow rather than the intended intermediate pressure-flow stimulus due to the lower pressure generating capacity at these higher lung volumes (McCool et al., 1995; McConnell et al., 2005). In contrast, participants in the TL-FRC group might have chosen to initiate breaths from slightly below FRC, thereby increasing pressure generating capacity, resulting in a true intermediate-pressure flow stimulus.

### General Considerations and Clinical Implications

The standard IMT protocol, TL-RV, did not provide an optimal loading as it did not increase PImax at higher lung volumes and did not increase V̇Imax (Figures 4, 5). Therefore, TL-RV at the intensities that were chosen by us and which are typically used in most other studies (30–50% PImax), actually resembles more a high pressure - low flow training instead of a training with intermediate flow and pressure rates (Tzelepis et al., 1994a, 1999; Romer and McConnell, 2003; McConnell and Romer, 2004; McConnell et al., 2005; Illi et al., 2012; HajGhanbari et al., 2013; Langer et al., 2015). TL training with inspirations initiated at a higher lung volume, FRC, might therefore be considered as a better alternative for TL training as it has the capacity to increase both PImax and V̇Imax over a larger range of lung volumes and especially at higher lung volumes. This supports an approach of performing TL at FRC at intensities of 40–50% relative to PImax assessed from FRC rather than initiating breaths from RV at similar intensities relative to PImax assessed from full expiration. The TFRL type of loading has the capacity to increase both PImax and V̇Imax over the largest range of lung volumes with large increases also on higher lung volumes and might therefore be regarded as an optimal training stimulus (Figures 4–6). An additional advantage of TFRL is that it provides data on training parameters that allows the clinician to control both quantity and quality of the training and provides feedback to the individual during unsupervised sessions.

To our knowledge, this is the first study to investigate both lung volume and flow specificity of IMT with intermediate flow and pressure rates. Improving the maximal inspiratory pressure generating capacity with IMT might be relevant for healthy subjects to overcome additional loads imposed on the respiratory pump during for example periods of loaded breathing or exercise hyperpnea (Rahn et al., 1946). Additionally, improving the maximal inspiratory flow further increases respiratory muscle power (Romer and McConnell, 2003; McConnell and Romer, 2004; Cormie et al., 2011). Increasing both parameters over the largest range of lung volumes and especially at higher lung volumes, might be functionally relevant during loaded breathing and exercise hyperpnea. The closer the training stimulus resembles the specific characteristics of a task the better the training outcome will be (Sharp, 1985; Hawley, 2008). Inspirations are initiated from FRC during resting breathing and during exercise the tidal volume and breathing frequency increase and inspirations are initiated from slightly below FRC. End-inspiratory lung volume during exercise is typically midway between FRC and TLC or even higher (Younes and Burks, 1985; Neder et al., 2003). Inspirations during exercise hyperpnea in highly trained athletes and patients with expiratory flow limitation such as in patients with chronic obstructive pulmonary disease are initiated at or above FRC and end-inspiratory lung volume will approach TLC (Johnson et al., 1992; Dempsey et al., 2008; McKenzie, 2012; O’Donnell et al., 2019). In addition, shortening velocity of the respiratory muscles has to increase during exercise hyperpnea (Prioux et al., 2000; Neder et al., 2003). Thus, TL-FRC and TFRL-RV provide a training stimulus to the inspiratory muscles at higher lung volumes (shorter muscle lengths) and at higher inspiratory flow (higher shortening velocities) which, corresponds to the operating muscle lengths and contraction patterns of the inspiratory muscles during exercise. Whether the observed differences in outcomes of the different IMT protocols will actually translate into improved breathing characteristics during exercise, in healthy, athletes and patients with exercise-induced expiratory flow limitation warrants further investigation.

### Strengths and Limitations

The recruitment procedure with posters and social media could have introduced a healthy volunteer effect (Delgado-Rodríguez and Llorca, 2004). While this did not introduce bias between the training groups, the observed improvements after IMT might be larger than in a sample from the general population. Additionally, two participants were lost to follow-up due to loss of interest to participate and declined to perform the post measurement. Nevertheless, the sample size of this study was sufficiently large based on the *a priori* power calculation and was three times the size of comparable studies (Tzelepis et al., 1994a, b, 1999; Romer and McConnell, 2003). The training program was largely home-based which reduced the level of control over the training interventions compared to a fully supervised training program, especially in the TL groups (O’Shea et al., 2007). By educating the volunteers at the start and at the weekly supervised training session, we aimed however to reduce variability in training performance. While the intervention period was rather short, it has been proven that effects of IMT can already be expected after only 4 weeks of training (HajGhanbari et al., 2013). The combination of a short home-based training period with a higher frequency (3 sessions per day) also resulted in a satisfactory adherence to the training. In the TL groups, the self-reported adherence to the training in the present study was high and comparable to the adherence reported in a previous study (Romer and McConnell, 2003). The adherence in the TFRL-RV group, objectively reported by the electronic training device, was lower. It must be pointed out however that self-reported adherence is often inaccurate and less reliable than objective measures of adherence (Rogliani et al., 2017). Adherence might therefore be overestimated in the TL-RV and TL-FRC group. A potential shortcoming of the experimental design is that we did not include a formal control group or a placebo group. However, sufficient data had already been collected in previous studies indicating that participants in control groups or sham groups achieved only very small improvements in inspiratory muscle strength or maximal inspiratory flow (Tzelepis et al., 1994a, b; Romer and McConnell, 2003; Illi et al., 2012).

## Conclusion

The present pilot study evaluated the principles of volume and flow specificity of a standard protocol (pressure threshold loading with inspirations initiated from residual volume) in comparison to two alternative IMT protocols. Our results provide initial evidence that improvements after using the standard protocol might be limited to increases in inspiratory muscle strength at lower lung volumes (between RV and FRC). We also observed that this standard protocol did not significantly improve maximal inspiratory flows against several external resistances at different lung volumes. Alternative approaches of IMT using either tapered flow resistive loading or pressure threshold loading initiated from functional residual capacity should be considered as potential alternatives for IMT. In our group of healthy volunteers both training protocols resulted in improvements in PImax and maximal inspiratory flows over a large range of lung volumes. Further research is warranted to investigate whether these findings can be confirmed in larger samples of both healthy subjects and patients. Whether the observed effects of these alternative IMT protocols will translate into larger improvements in respiratory muscle endurance, respiratory muscle function during whole body exercise, and exercise capacity also warrants further investigation.

## Data Availability Statement

The raw data supporting the conclusions of this article will be made available by the authors upon request.

## Ethics Statement

The studies involving human participants were reviewed and approved by Ethics Committee Research UZ/KU Leuven. The patients/participants provided their written informed consent to participate in this study.

## Author Contributions

RG and DL contributed to conception and design of the study. DL and MV performed the data collection. MV organized the database, performed the statistical analysis, and wrote the first draft of the manuscript. All authors contributed to manuscript revision, read, and approved the submitted version.

## Conflict of Interest

DL acknowledges receiving compensation for travel costs from HaB International for participating as an invited speaker in the 27th Annual Meeting of the Japan Society for Respiratory Care and Rehabilitation, November 17-18, 2017 in Sendai, Japan and related educational activities in Tokyo, Japan that were promoted by POWERbreathe International. The POWERbreathe KHP2 devices, used in this study were the same devices that our group used in the multicentre RCT published in 2018 (https://pubmed.ncbi.nlm.nih.gov/29914940/). The company, HAB – POWERbreathe International Ltd., (Warwickshire, England), kindly offered us to keep using those devices for our clinical care and research projects after completion of this previous study. The pressure threshold loading devices were not provided by the company but purchased by us through their local retailer. The consumables for the electronic devices (valve heads and mouthpieces) were purchased by our research group through their local retailer. The remaining authors declare that the research was conducted in the absence of any commercial or financial relationships that could be construed as a potential conflict of interest.
