# Supplementary material for: Training Specificity of Inspiratory Muscle Training Methods: A Randomized Trial
Source: Front Physiol. 2020 Dec 3;11:576595. doi: 10.3389/fphys.2020.576595 (PMC7744620; doi:10.3389/fphys.2020.576595)
Supplement: Supplementary file 1 [file Table_1.DOCX]

Supplementary Tables

| **Table 1: Within group comparison of increase in maximal inspiratory flow** | | | | | | | | | | | | | | | |
| --- | --- | --- | --- | --- | --- | --- | --- | --- | --- | --- | --- | --- | --- | --- | --- |
| **Load (% PImax baseline)** | **No external load** | | | **20% PImax** | | | **30% PImax** | | | **50% PImax** | | | **70% PImax** | | |
| **Max. insp. Flow (L/s)** | **Pre** | **Post** | **P-value** | **Pre** | **Post** | **P-value** | **Pre** | **Post** | **P-value** | **Pre** | **Post** | **P-value** | **Pre** | **Post** | **P-value** |
| **TL-RV** |  |  |  |  |  |  |  |  |  |  |  |  |  |  |  |
| RV | 5.4 ± 1.1 | 5.7±1.1 | 0.39 | 4.5±1.0 | 4.6±0.8 | 0.40 | 3.9 ± 1.0 | 4.2 ± 1.0 | 0.39 | 2.8 ± 0.6 | 3.2 ± 0.9 | 0.19 | 1.9 ± 0.4 | 2.4 ± 0.9 | 0.10 |
| FRC | 5.3±1.5 | 5.5±1.1 | 0.84 | 4.2±1.3 | 4.4±1.2 | 0.84 | 3.9 ± 1.3 | 3.9 ± 1.0 | 0.91 | 2.9 ± 1.2 | 2.8 ± 1.0 | 0.91 | 1.7 ± 0.8 | 2.2 ± 0.9 | 0.42 |
| 1/2 IC | 4.5±1.3 | 4.8±1.2 | 0.75 | 3.5±1.1 | 3.6±0.9 | 0.89 | 3.0 ± 1.1 | 3.2 ± 0.8 | 0.87 | 2.5 ± 1.3 | 2.6 ± 0.7 | 0.97 | 1.8 ± 1.1 | 1.8 ± 0.9 | 0.97 |
| **TFRL-RV** |  |  |  |  |  |  |  |  |  |  |  |  |  |  |  |
| RV | 5.5±1.3 | 6.9±2.5 | **<0.001** | 4.8±1.2 | 5.5±1.6 | **0.03** | 4.0 ± 1.0 | 5.0 ± 1.3 | **0.007** | 2.8 ± 0.8 | 3.8 ± 0.9 | **0.007** | 2.0 ± 0.8 | 2.8 ± 0.8 | **0.03** |
| FRC | 5.5±1.2 | 7.1±2.5 | **<0.001** | 4.4±1.1 | 5.8±1.9 | **<0.001** | 4.1 ± 1.1 | 5.3 ± 1.5 | **<0.001** | 3.1 ± 0.9 | 4.1 ± 1.3 | **0.004** | 2.0 ± 0.8 | 3.0 ± 1.2 | **0.004** |
| 1/2 IC | 4.8±1.3 | 6.0±1.7 | **<0.001** | 3.6±0.9 | 4.4±1.2 | **0.004** | 3.2 ± 1.3 | 4.0 ± 1.2 | **0.003** | 2.4 ± 1.2 | 3.5 ± 1.2 | **<0.001** | 1.8 ± 1.1 | 2.6 ± 0.9 | **0.003** |
| **TL-FRC** |  |  |  |  |  |  |  |  |  |  |  |  |  |  |  |
| RV | 5.3±1.2 | 5.7±1.2 | 0.24 | 4.2±1.2 | 4.8±1.0 | **0.03** | 3.9 ± 1.0 | 4.3 ± 1.0 | 0.24 | 2.6 ± 0.9 | 3.6 ± 0.8 | **<0.001** | 1.9 ± 1.4 | 2.6 ± 0.7 | **0.01** |
| FRC | 5.1±1.2 | 5.8±1.0 | **<0.001** | 4.2±1.1 | 4.8±1.0 | **<0.001** | 3.7 ± 1.0 | 4.5 ± 1.1 | **<0.001** | 2.8 ± 1.0 | 3.8 ± 1.1 | **<0.001** | 2.0 ± 0.9 | 2.9 ± 1.0 | **<0.001** |
| 1/2 IC | 4.6±1.1 | 5.1±1.0 | 0.003* | 3.3±1.0 | 4.0±1.0 | **0.001** | 3.0 ± 1.2 | 3.7 ± 0.9 | **0.001** | 2.3 ± 1.0 | 3.1 ± 1.0 | **<0.001** | 1.4 ± 0.9 | 2.3 ± 1.0 | **<0.001** |
| Pre and post measurements of maximal inspiratory flow on three different lung volumes measured against external loads performed with a pressure threshold loading: no load, 20, 30, 50 and 70% of the PImax baseline: maximal inspiratory strength at baseline. All measurements were performed on three lung volumes, RV: Residual volume, FRC: functional residual capacity and 1/2 IC: midway between FRC and total lung capacity. Data are presented as mean ± SD. Max. insp. Flow: maximal inspiratory flow, TL-RV (n=15): Training protocol performed with the pressure threshold loading with inspirations initiated from residual volume, TFRL-RV (n=15): Training protocol performed with the tapered flow resistive loading with inspirations initiated from residual volume, TL-FRC (n=16): Training protocol performed with the pressure threshold loading with inspirations initiated from functional residual capacity. | | | | | | | | | | | | | | | |

| **Table 2: Within group comparison of inspiratory volume during resistive breathing manoeuvres** | | | | | | | | | | | | | | | |
| --- | --- | --- | --- | --- | --- | --- | --- | --- | --- | --- | --- | --- | --- | --- | --- |
| **Load (% PImax baseline)** | **No external load** | | | **20% PImax** | | | **30% PImax** | | | **50% PImax** | | | **70% PImax** | | |
| **Inspiratory volume (L)** | **Pre** | **Post** | **P-value** | **Pre** | **Post** | **P-value** | **Pre** | **Post** | **P-value** | **Pre** | **Post** | **P-value** | **Pre** | **Post** | **P-value** |
| **TL-RV** |  |  |  |  |  |  |  |  |  |  |  |  |  |  |  |
| RV | 3.5 ± 1.0 | 3.3 ± 0.8 | 0.85 | 3.0 ± 0.7 | 2.9 ± 0.6 | 0.90 | 2.7 ± 0.6 | 2.4 ± 0.5 | 0.52 | 2.0 ±0.5 | 1.9 ± 0.5 | 0.90 | 1.7 ± 0.6 | 1.7 ± 0.7 | 0.90 |
| FRC | 2.6 ± 0.8 | 2.5 ± 0.5 | 0.57 | 2.3 ± 0.7 | 2.0 ± 0.4 | 0.55 | 2.1 ± 0.7 | 1.9 ± 0.5 | 0.55 | 1.8 ± 1.0 | 1.5 ± 0.5 | 0.15 | 1.3 ± 0.6 | 1.2 ± 0.4 | 0.57 |
| 1/2 IC | 1.6 ± 0.6 | 1.5 ± 0.6 | 0.74 | 1.3 ± 0.6 | 1.2 ± 0.3 | 0.74 | 1.3 ± 0.7 | 1.1 ± 0.3 | 0.68 | 1.1 ± 0.7 | 0.9 ± 0.3 | 0.74 | 1.0 ± 0.6 | 0.8 ± 0.2 | 0.68 |
| **TFRL-RV** |  |  |  |  |  |  |  |  |  |  |  |  |  |  |  |
| RV | 4.1 ± 1.5 | 4.1 ± 1.4 | 0.97 | 3.8 ± 1.3 | 3.7 ± 1.4 | 0.97 | 3.5 ± 1.3 | 3.4 ± 1.3 | 0.97 | 2.8 ± 1.0 | 3.0 ± 1.1 | 0.77 | 2.2 ± 1.2 | 2.2 ± 0.9 | 0.97 |
| FRC | 3.0 ± 1.1 | 3.1 ± 1.1 | 0.86 | 2.7 ± 1.0 | 2.7 ± 1.0 | 0.89 | 2.6 ± 0.9 | 2.5 ± 0.8 | 0.86 | 2.3 ± 0.8 | 2.2 ± 0.8 | 0.86 | 2.0 ± 0.8 | 1.8 ± 0.6 | 0.63 |
| 1/2 IC | 1.6 ± 0.5 | 1.7 ± 0.4 | 0.61 | 1.3 ± 0.3 | 1.4 ± 0.4 | 0.61 | 1.2 ± 0.4 | 1.4 ± 0.6 | 0.39 | 1.1 ± 0.4 | 1.3 ± 0.4 | 0.39 | 1.0 ± 0.4 | 1.1 ± 0.3 | 0.53 |
| **TL-FRC** |  |  |  |  |  |  |  |  |  |  |  |  |  |  |  |
| RV | 3.7 ± 1.0 | 3.8 ± 0.8 | 0.79 | 3.3 ± 0.9 | 3.3 ± 0.8 | >0.99 | 3.0 ± 0.8 | 3.0 ± 0.8 | 0.95 | 2.4 ± 0.9 | 2.5 ± 0.6 | 0.79 | 1.8 ± 0.9 | 2.1 ± 0.8 | **0.03** |
| FRC | 2.9 ± 0.7 | 2.9 ± 0.7 | 0.57 | 2.4 ± 0.6 | 2.5 ± 0.6 | 0.36 | 2.2 ± 0.6 | 2.3 ± 0.6 | 0.36 | 1.8 ± 0.6 | 2.0 ± 0.6 | **0.02** | 1.5 ± 0.6 | 1.7 ± 0.5 | **0.01** |
| 1/2 IC | 1.7 ± 0.6 | 1.8 ± 0.5 | 0.24 | 1.3 ± 0.3 | 1.5 ± 0.5 | 0.17 | 1.3 ± 0.4 | 1.5 ± 0.4 | 0.17 | 1.1 ± 0.3 | 1.4 ± 0.5 | 0.12 | 0.9 ± 0.3 | 1.1 ± 0.4 | 0.17 |
| Pre and post measurements of inspiratory volume during resistive breathing maneouvres on three different lung volumes measured against external loads performed with a pressure threshold loading: no load, 20, 30, 50 and 70% of the PImax baseline: maximal inspiratory strength at baseline. All measurements were performed on three lung volumes, RV: Residual volume, FRC: functional residual capacity and 1/2 IC: midway between FRC and total lung capacity. Data are presented as mean ± SD. TL-RV (n=15): Training protocol performed with the pressure threshold loading with inspirations initiated from residual volume, TFRL-RV (n=15): Training protocol performed with the tapered flow resistive loading with inspirations initiated from residual volume, TL-FRC (n=16): Training protocol performed with the pressure threshold loading with inspirations initiated from functional residual capacity. | | | | | | | | | | | | | | | |

| **Table 3: Within group comparison of the increase in maximal inspiratory flow** | | | |
| --- | --- | --- | --- |
| **groups** | **Mean difference (L/s)** | **SE of difference (L/s)** | **p-value** |
| **TL-RV** |  |  |  |
| RV vs FRC | 0.19 | 0.30 | 0.87 |
| RV vs 1/2 IC | 0.21 | 0.30 | 0.87 |
| FRC vs 1/2 IC | 0.01 | 0.30 | 0.96 |
| **TFRL-RV** |  |  |  |
| RV vs FRC | -0.28 | 0.09 | **0.01** |
| RV vs 1/2 IC | 0.01 | 0.09 | 0.92 |
| FRC vs 1/2 IC | 0.29 | 0.09 | **0.01** |
| **TL-FRC** |  |  |  |
| RV vs FRC | -0.17 | 0.28 | 0.91 |
| RV vs 1/2 IC | -0.11 | 0.28 | 0.91 |
| FRC vs 1/2 IC | 0.06 | 0.28 | 0.91 |
| The increase in maximal inspiratory flow in litre per second (L/s) after the training period is compared within the training groups between the three different lung volumes. Mean difference with the standard error (SE) of difference is presented as the increase in maximal inspiratory flow of the 1^st^ lung volume minus the 2^nd^ lung volume. RV: residual volume, FRC: functional residual capacity and 1/2 IC: midway between FRC and total lung capacity. 3 training groups were compared on these lung volumes, TL-RV (n=15): Training protocol performed with the pressure threshold loading with inspirations initiated from residual volume, TFRL-RV (n=15): Training protocol performed with the tapered flow resistive loading with inspirations initiated from residual volume, TL-FRC (n=16): Training protocol performed with the pressure threshold loading with inspirations initiated from functional residual capacity. | | | |

| **Table 4: Between group comparison of increase in maximal inspiratory flow** | | | |
| --- | --- | --- | --- |
| **(Group 1 – group 2)** | **Mean difference (L/s)** | **SE of difference (L/s)** | **p-value** |
| **TL-RV vs TFRL RV** |  |  |  |
| RV | -0.64 | 0.30 | **0.04** |
| FRC | -1.08 | 0.36 | **<0.01** |
| 1/2 IC | -0.80 | 0.30 | **0.01** |
| **TL-RV vs TL-FRC** |  |  |  |
| RV | -0.25 | 0.22 | 0.25 |
| FRC | -0.61 | 0.25 | **0.02** |
| 1/2 IC | -0.57 | 0.25 | **0.03** |
| **TFRL-RV vs TL-FRC** |  |  |  |
| RV | 0.39 | 0.28 | 0.18 |
| FRC | 0.47 | 0.28 | 0.10 |
| 1/2 IC | 0.24 | 0.25 | 0.35 |
| The increase in maximal inspiratory flow in litre per second (L/s) after the training period is compared between the three groups. The mean difference between the groups are depicted in the table with the standard error (SE) of difference. Mean difference is presented as the increase in maximal inspiratory flow of the 1^st^group minus the 2^nd^ group. Difference on three lung volumes were compared, RV: residual volume, FRC: functional residual capacity and 1/2 IC: midway between FRC and total lung capacity. 3 training groups were compared on these lung volumes, TL-RV (n=15): Training protocol performed with the pressure threshold loading with inspirations initiated from residual volume, TFRL-RV (n=15): Training protocol performed with the tapered flow resistive loading with inspirations initiated from residual volume, TL-FRC (n=16): Training protocol performed with the pressure threshold loading with inspirations initiated from functional residual capacity. | | | |
